# Contact-aware multi-skill learning framework with hybrid force-motion control for stability and force regulation in robotic physiotherapy

**DOI:** 10.3389/frobt.2026.1892296

**Published:** 2026-08-06

**Authors:** Xueqian Zhai, Qihao Feng, Haochen Zheng, Yi Luo, Huikun Hao, An Yan, Hongmin Wu

**Affiliations:** 1 College of Engineering, Shandong Xiehe University, Jinan, China; 2 Institute of Intelligent Manufacturing, Guangdong Academy of Sciences, Guangzhou, China

**Keywords:** compliant control, contact-aware, force regulation, robot skill learning, robotic physiotherapy

## Abstract

Robot-assisted physiotherapy has attracted increasing attention for its potential to provide repeatable, stable, and controllable physical interaction during rehabilitation-oriented therapy. However, contact-rich physiotherapy tasks remain challenging because the robot must reproduce therapist-demonstrated massage skills while adapting to non-planar and deformable body surfaces, suppressing impact during contact transition, and maintaining stable force regulation. This paper proposes a contact-aware robot-assisted physiotherapy framework that integrates task-space skill generalization, contact state estimation, dynamic velocity adjustment, force-error compensation, and bounded variable impedance control. Therapist-guided demonstrations are encoded using Dynamic Movement Primitives (DMPs) to construct a physiotherapy skill library, enabling typical massage skills, including kneading, patting, pushing, and pressing, to be generalized to new start and goal points in the robot Cartesian task space. During execution, force/torque feedback is used to estimate the contact point and surface normal, update the local task frame, regulate the approach velocity, and compensate for force-tracking errors. Experimental validation was conducted on a silicone abdominal model and in a preliminary healthy-volunteer back physiotherapy test. The results show that the proposed dynamic contact strategy suppresses excessive impact during contact transition, avoiding the 165.86 N peak impact observed under high-speed contact. The force compensation strategy reduces the force-tracking RMSE from 0.835 N to 0.395 N , corresponding to a 52.69% improvement. In addition, the bounded variable impedance strategy improves motion-force coordination compared with fixed-stiffness control. These results demonstrate that the proposed framework improves contact transition safety, force regulation accuracy, and task adaptability at the control-performance level, providing a feasible basis for further development of robotic physiotherapy systems.

## 1 Introduction

With the rapid advancement of science and technology, the field of robotic technology has made remarkable strides, particularly within industrial manufacturing. However, the utilization of robots in medically assisted scenarios, particularly in human rehabilitation, remains in its nascent stages (Garcia-Gonzalez et al., 2022). Unlike industrial applications that mainly prioritize efficiency and precision, medical-assistance robots must additionally consider individual differences, physiological variations, safety constraints, and treatment efficacy during physical human-robot interaction (Molle et al., 2025). This complexity underscores the unique challenges faced when integrating robotics into healthcare settings, where the wellbeing of patients is paramount.

Currently, robot-assisted rehabilitation and treatment have yielded some encouraging outcomes (Payedimarri et al., 2022; Ai et al., 2023; Yang et al., 2023). For instance, robots are capable of providing feasible motion control and force adjustment in the field of rehabilitation, aiding patients in physical therapy (Tamantini et al., 2022). Previously, we conducted research on the rehabilitation of human upper limbs (Wu et al., 2022), focusing on skill learning as a reference point. We introduced a framework for skill representation, learning, and modulation of rehabilitation-assisted robots, utilizing manifold mapping and Gaussian process techniques. The relevant methods have been recognized by the rehabilitation department of hospitals and integrated into the rehabilitation robot system for application. However, further research and development are necessary to enhance the application effectiveness of robots in human rehabilitation and disease treatment (Nizamis et al., 2021). This includes improvements in robot control algorithms, the development of individualized treatment plans, and the training and utilization of healthcare therapists in robotics technology.

Despite these advances, contact-rich robotic physiotherapy still faces several key challenges. First, many learning-from-demonstration methods mainly focus on reproducing demonstrated trajectories, while their adaptation to different body surfaces, contact regions, and patient-specific geometries remains limited. Second, conventional fixed-parameter impedance or force control strategies are often insufficient for deformable and non-planar human body surfaces, where the contact point, surface normal, and tissue deformation may change during execution. Third, contact transition, force regulation, and motion tracking are frequently treated as separate problems, which may lead to excessive impact, unstable force tracking, or insufficient motion-force coordination during therapy. These limitations motivate the development of a contact-aware framework that integrates task-space skill generalization, contact state estimation, and hybrid force-motion control for robotic physiotherapy.

The objective of this work is to develop a contact-aware robotic physiotherapy framework that can reproduce therapist-demonstrated massage skills while maintaining stable and safe force interaction on deformable human-like surfaces. The main contributions are summarized as follows:

- A Dynamic Movement Primitive (DMP)-based physiotherapy skill library is constructed from therapist-guided demonstrations, enabling task-space generalization of typical massage skills, including pressing, patting, kneading, and pushing.
- A contact point and surface normal estimation strategy is integrated into the execution loop, allowing the robot to update the local task frame when interacting with curved and deformable human-like surfaces.
- A hybrid force-motion control strategy is proposed by coupling dynamic velocity regulation, bounded variable impedance scheduling, and force-error compensation, which improves contact transition safety and force regulation accuracy.
- The framework is experimentally validated on a silicone model and healthy-subject back physiotherapy tasks, including pressing, patting, kneading, and pushing skills, with quantitative comparisons against fixed-speed, no-compensation, and fixed-stiffness baselines.

## 2 Related work

In recent years, Learning from Demonstration (LfD) (Ravichandar et al., 2020) has emerged as a straightforward and intuitive approach to instruct robots in task completion through human-computer physical interaction and recording of status information (Huang et al., 2021). However, when faced with diverse task objects or the need for cross-domain generalization, traditional teaching-learning methods necessitate retraining, leading to inadequate generalization and environmental adaptability (Zhang et al., 2022). For robot-assisted physiotherapy, this limitation is particularly important because the robot needs to reproduce therapist-demonstrated skills while adapting to different body surfaces, contact regions, and patient-specific task requirements.

To learn massage skills, therapists demonstrated robot actions through physical human-computer interaction, obtaining the demonstration dataset (Stepputtis et al., 2020). Several methods are available for learning from demonstrations, including Hidden Markov Model (HMM) (Ali and Bouguila, 2020), Gaussian Process (GP) (Jaquier et al., 2020), Gaussian Mixture Model (GMM) (Ye et al., 2022), Stable estimator of dynamical systems (SEDS) (Si et al., 2021), and more. Among these methods, DMP (Zhang Y. et al., 2021) provides a compact representation for encoding demonstrated trajectories and can quickly adapt the learned motion to new start and goal points. Consequently, DMPs are suitable for constructing a physiotherapy skill library in which typical massage skills can be generalized within the robot Cartesian task space (Zhai et al., 2024). It should be noted that, in this work, skill learning refers to learning from therapist-guided demonstrations in the robot task space, rather than cross-embodiment transfer from human arm kinematics to robot kinematics. In contrast, other methods heavily depend on teaching experiences and specific teaching domains, which hinders their quick migration to unknown feasible areas. Consequently, they may struggle to meet the diverse and personalized needs of patients during the treatment process (Chan et al., 2017).

On the other hand, in the process of interacting with patients, robots need to execute dynamic compliant control (Peng et al., 2022) due to the characteristics of human skin tissue (Saeidi et al., 2022). Fixed impedance parameter control is inadequate as it cannot accurately perceive changes in the surrounding environment, increasing the complexity of the control system and often failing to meet the compliance required by the task (El Zaatari et al., 2019). Introducing a variable impedance control strategy allows for real-time adjustment of impedance parameters to balance motion accuracy and flexibility control (Spyrakos-Papastavridis and Dai, 2021). Zeng et al. (2024) developed a LfD framework that introduced a hierarchical torque controller for achieving compliant trajectory tracking and maneuverability control. The framework incorporates an optimization-based force control mechanism to replicate the desired force profile. Luo et al. (2024) proposed a physical human-robot interaction framework that minimizes interaction forces and improves trajectory tracking accuracy through adaptive trajectory adjustment and impedance control based on surface electromyography signals. This method offers more precise control and ensures a safer contact environment. Recent studies have also emphasized the importance of adaptive interaction control and quantitative evaluation in robot-assisted rehabilitation systems (Molle et al., 2026). These works indicate that stable force regulation, contact safety, and individualized interaction strategies are essential for rehabilitation-oriented physical human-robot interaction.

Although previous studies have achieved promising progress in LfD, impedance control, and robot-assisted rehabilitation, several limitations remain for contact-rich physiotherapy tasks. First, many learning-based methods mainly focus on reproducing demonstrated trajectories, while their adaptation to different body surfaces and contact regions remains limited. Second, conventional fixed-parameter impedance controllers are insufficient for deformable and non-planar human body surfaces, where the contact state and surface normal may change during execution. Third, contact transition is often treated separately from force regulation, which may lead to excessive impact or unstable force tracking. Finally, existing evaluations often report isolated results for trajectory tracking or force control, but lack integrated metrics for assessing the overall performance of robot-assisted physiotherapy. These limitations motivate the proposed contact-aware multi-skill learning and hybrid force-motion control framework.

**FIGURE 1:**
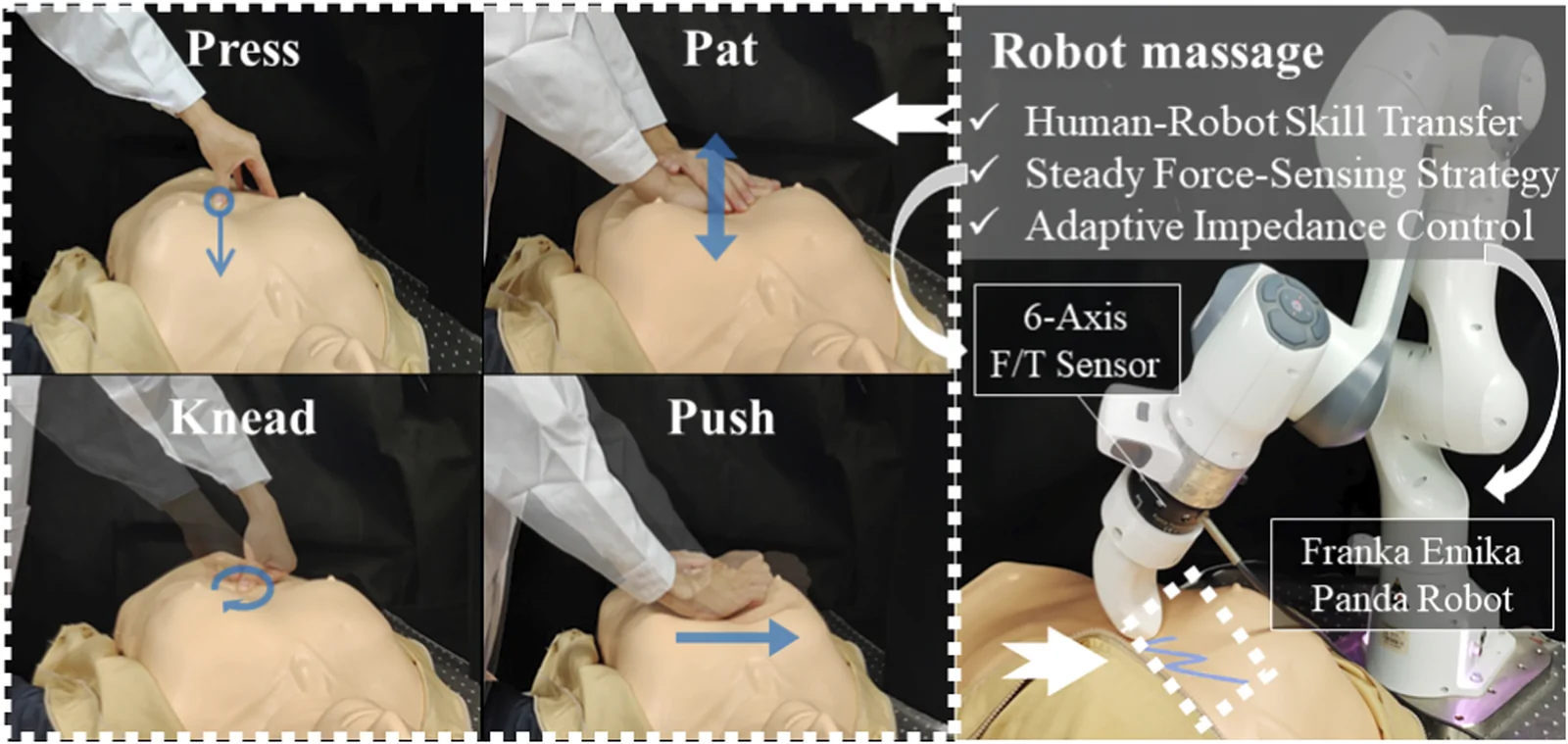
Therapists often select different massage skills (Zhang X. et al., 2021) based on symptoms and experience. However, ensuring the accuracy and consistency of treatment for different patients can be challenging (Shahid et al., 2023). Robot assistance provides a promising solution to these issues. By learning from therapist-guided demonstrations in the robot task space, robots can reproduce typical massage skills and generalize them to new start and goal points in a stable and contact-aware manner (Yang et al., 2019).

The objective of this work is to develop a contact-aware robotic physiotherapy framework that can reproduce therapist-demonstrated massage skills while maintaining stable and safe force interaction on deformable human-like surfaces, as shown in Figure 1. Unlike methods that only reproduce demonstrated trajectories or apply fixed-parameter impedance control, the proposed framework couples the learned motion primitive with online contact-state estimation and force-error-aware impedance regulation. This coupling is particularly important for physiotherapy tasks because the robot must maintain stable contact force while moving over non-planar and deformable body surfaces. Specifically, DMP-based task-space skill generalization provides the nominal motion reference, contact point and surface normal estimation update the local task frame, and dynamic velocity adjustment, force-error compensation, and bounded variable impedance control jointly improve contact transition safety and force regulation accuracy.

By addressing these key issues, this research aims to contribute to the advancement of robot-assisted physiotherapy, with a focus on control-level contact stability, force regulation accuracy, and task adaptability rather than clinical efficacy validation. In Section 3, we elaborate on the proposed framework, including skill learning, contact estimation, dynamic velocity adjustment, force regulation, and hybrid force-motion control. Section 4 evaluates the performance of the robot when performing four massage skills, including kneading, patting, pushing, and pressing. Section 5 summarizes the proposed framework, discusses its limitations, and outlines future work.

## 3 Methods

### 3.1 Problem statement and framework illustration

Traditional massage skills often require therapists to invest significant time and energy in addressing various challenges (Wei et al., 2016). Patients with different diseases may require different adjustment plans based on their body types. Even patients with the same disease may exhibit variations, necessitating therapists to employ different massage skills (Foley et al., 2020). Moreover, different therapists utilizing distinct skills can yield varying outcomes, introducing treatment uncertainties, and exacerbating the shortage of medical staff (Javaid et al., 2022).

**FIGURE 2:**
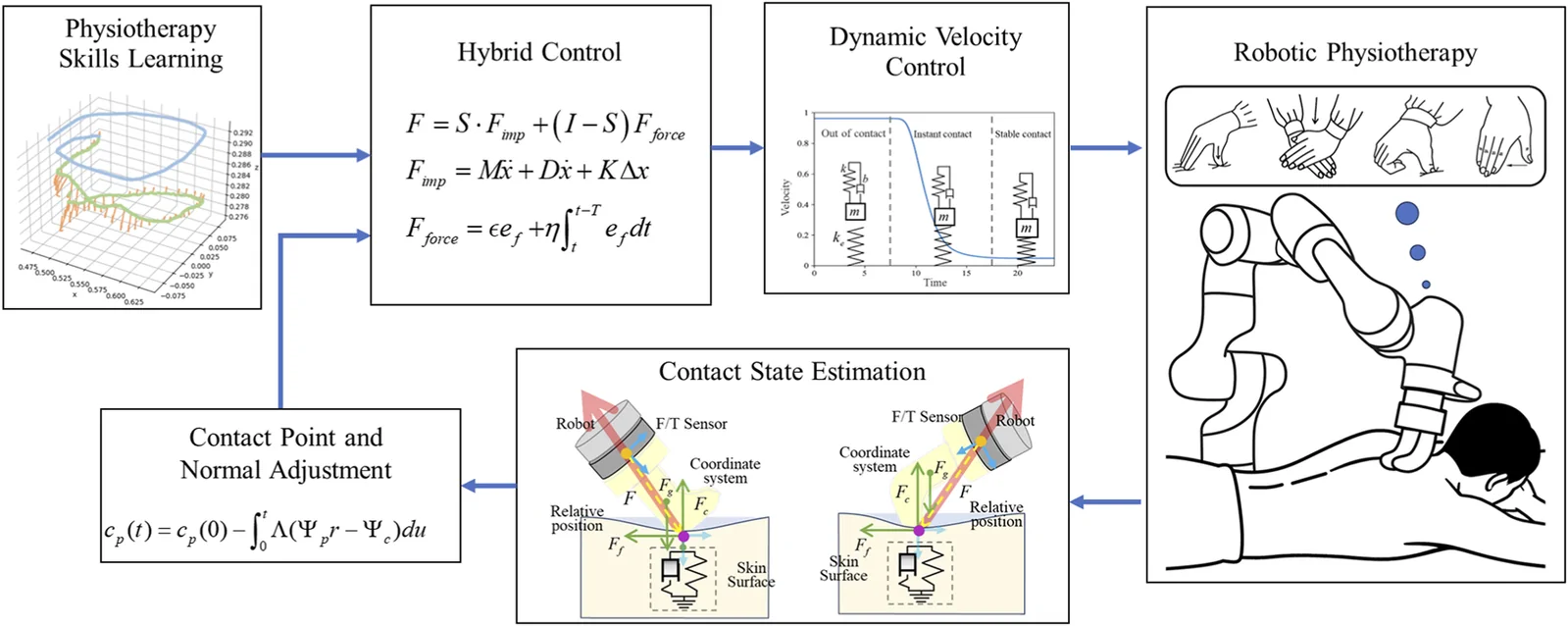
Overview of the proposed contact-aware robotic physiotherapy framework. The DMP-based skill learning module generates a task-space reference trajectory, while force/torque feedback is used for contact point and surface normal estimation, dynamic velocity adjustment, force-error compensation, and bounded variable impedance control, enabling stable and constant-force interaction during robotic physiotherapy.

As shown in Figure 2, the design concepts of our proposed framework include four main aspects. Firstly, through therapist-guided demonstration learning, efficient acquisition and task-space generalization of skills can be achieved, which reduces the size of the training set and enhances the efficiency of training. The requirement for generalization information is minimal, thereby shortening the time required for generalization. A repository of robotic massage skills has been established based on a variety of robotic massage techniques. Secondly, by utilizing contact point estimation and normal adjustment, the system adapts to different massage subjects, thereby improving the quality of the massage. Subsequently, a dynamic velocity adjustment strategy is implemented to safely and stably approach the contact point, enhancing the safety of robotic collaboration. Lastly, due to the non-planar nature of the human body surface and its certain degree of deformability, a real-time feedback force regulation strategy has been proposed. This strategy enables the robot to exert the anticipated force stably.

The modules in the proposed framework are not executed independently. The DMP module first generates a nominal task-space trajectory according to the selected massage skill and the specified start/goal points. During execution, the contact point and surface normal estimated from the force/torque (F/T) sensor are used to update the local task frame and align the DMP-generated trajectory with the patient surface. The dynamic velocity strategy regulates the approach velocity before and after contact to suppress impact. The generalized trajectory also provides temporal and spatial information for stiffness scheduling, while the measured force error is used for force compensation along the estimated surface normal. In this way, the motion reference, contact estimation, variable impedance, and force regulation are integrated into a closed-loop hybrid force-motion control framework.

### 3.2 Learning and generalization of massage skills

**FIGURE 3:**
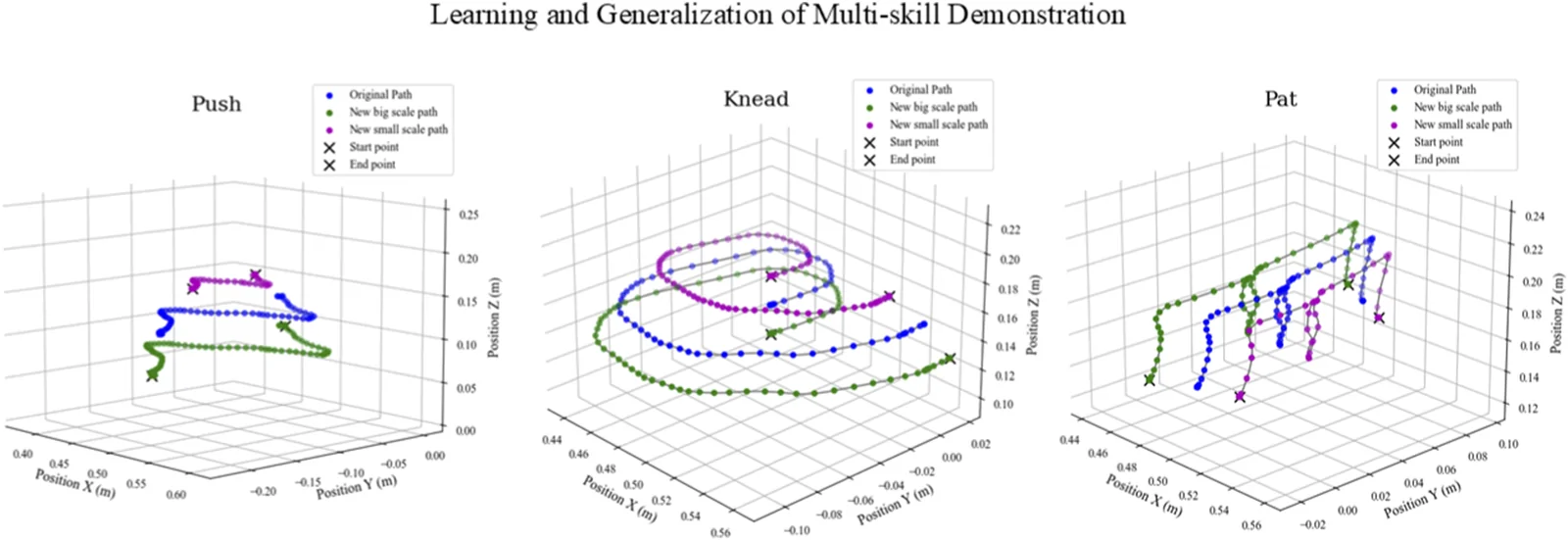
Teaching trajectories and generalized results of three massage skills: kneading, pushing, and patting. The blue curve denotes the original demonstration, while the green and purple curves denote spatially generalized trajectories with different scales.

In the process of massage, therapists automatically adjust the massage points and paths based on individual requirements (Pang et al., 2019). We expect a robot to achieve self-adaptation for a new requirement by only needing to obtain the start and end endpoint information on the contact surface’s target area. Therefore, DMP method is considered in the paper (Cui et al., 2022). A DMP can be regarded as consisting of a transformation system that generates the desired trajectory and a canonical system that controls the phase evolution of the movement, as described in Equations 1 and 2. The discrete DMP model is defined as follows:

$$\tau_{t}\dot{z}=\alpha_{z}\left(\beta_{z}\left(g-y\right)-\tau_{t}z\right)+g_{f}\left(x\right)\left(g-y_{0}\right)f_{s}\left(x\right)$$ (1)

$$\tau_{t}\dot{y}=z$$ (2)

where y , $\dot{y}$ is the position and velocity, g the target position, $y_{0}$ the initial position, and the phase variable x is used to avoid direct dependence on time, and $\tau_{t}$ is usually the normal number of time factors, which are generally set to the total duration of the movement, $T=t_{f}-t_{0}$ , $t_{0}$ is the initial time and $t_{f}$ the final time. During execution, the trajectory goal of the DMP is dynamically adjusted based on the estimated contact point, as shown in Figure 3. This ensures continuous alignment between the robot’s operational plane for therapeutic tasks and the patient’s surface anatomy, thereby enabling real-time adaptation of therapeutic movements.

For each physiotherapy skill, such as pushing, kneading, patting, or pressing, an independent DMP primitive is constructed. The nonlinear forcing term encodes the characteristic geometric pattern of each skill, while the start and goal states provide spatial modulation for patient-specific execution. In the current implementation, a representative demonstration is selected for each skill to construct the skill library, and multiple demonstrations are not fused probabilistically in the DMP weight space.

In this work, the skills are learned from therapist-guided demonstrations in the robot Cartesian task space. The demonstrated massage trajectories are encoded as DMPs and can be spatially generalized to new start and goal points on different contact regions. Therefore, the proposed framework does not address cross-embodiment transfer from human arm kinematics to robot kinematics. Instead, it focuses on intra-robot task-space skill generalization and contact-aware adaptation during execution.

### 3.3 Contact interaction strategy

#### 3.3.1 Unknown surface contact point and normal estimation

For a robot to perform physiotherapy, it needs to replicate the skills of a physiotherapist by adopting different postures to apply the desired force effectively Dragusanu et al. (2022). The robot’s massage end-effector is designed to mimic the human thumb, and different end gestures correspond to various massage skills to achieve improved treatment outcomes Li et al. (2020).

Surface contact point estimation plays a crucial role in identifying the spatial information of the contact point accurately, even in different postures (Chen et al., 2021). This capability facilitates subsequent treatment adjustments and ensures stable contact throughout the therapy session. Additionally, normal estimation involves real-time adjustment of the end-effector’s posture based on the perceived external force in the direction of free movement (Li et al., 2020). This adaptive approach allows the robot to effectively navigate non-rigid surfaces, ensuring optimal therapy delivery (Nocentini et al., 2022).

**FIGURE 4:**
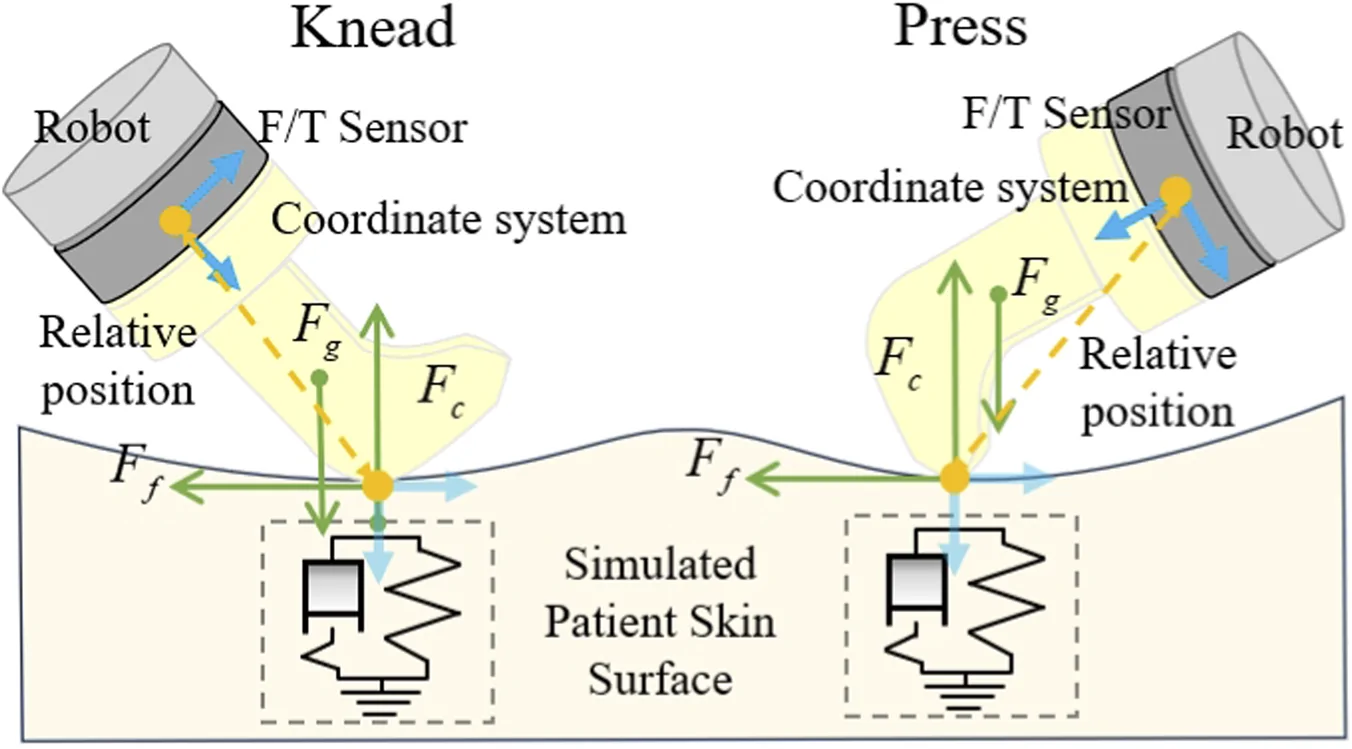
Contact force analysis between the robot end-effector and the human-body model. $F_{c}$ , $F_{g}$ , and $F_{f}$ denote the deformation force, gravity component, and friction force, respectively.

To estimate the position of the contact point, $p_{c}$ , where $p_{c}=p_{e}+r$ and $p_{e}$ represents the position of the robot’s end effector, the relative position r from the actuator’s end to the tool’s end must be clearly defined, as shown in the contact state estimation section in Figure 4. The rotation of the robot’s end effector affects the relative position r , while the relative position $c_{p}$ , with the robot’s end as the origin, remains unaffected. This is expressed as $c_{p}=R^{⊤}r$ , where R denotes the orientation of the robot’s end effector. Based on the feedback from the torque sensor, the measured force $f_{s}$ and moment $\tau_{s}$ of the robot are known, along with the gravitational force $f_{g}$ acting on the tool and the position of the tool’s center of mass relative to the end effector, $p_{g}$ . Consequently, the measured force and moment at the tool’s end are given by $f_{m}=f_{s}-f_{g}$ and $\tau_{m}=\tau_{s}-p_{g}\times f_{g}$ , respectively. The dynamic update strategy for the relative position between the end effector and the contact point is:

$$\begin{array}{c} c_{p}\left(t\right)=c_{p}\left(0\right)-\int_{0}^{t}\Lambda \left(\Psi_{p}\left(u\right)c_{p}\left(u\right)-\Psi_{c}\left(u\right)\right)du \end{array}$$ (3)

In Equation 3, the $c_{p}\left(0\right)$ denotes the relative position of the initial contact point, Λ is a positive definite matrix that regulates the convergence rate and stability of the estimation process. It minimizes the error between the current estimated contact point position and the desired position, expressed as $\Psi_{p}c_{p}-\Psi_{c}$ . Here, $\Psi_{p}$ is the dynamic matrix that captures the influence of contact forces on the contact point’s position, and its update strategy is defined as follows:

$$\begin{array}{c} \Psi_{p}\left(t\right)=\Psi_{p}\left(0\right)+\int_{0}^{t}\left(-\xi \Psi_{p}\left(u\right)+H\left(f_{m}\left(u\right)\right)\right)du \end{array}$$ (4)

where $\Psi_{p}\left(0\right)$ represents the initial dynamic matrix as a zero matrix, and ξ controls the update speed. In Equation 4, the term $H\left(f_{m}\left(u\right)\right)={\left\|f_{m}\left(u\right)\right\|}^{2}I_{d}-f_{m}\left(u\right)f_{m}^{⊤}\left(u\right)$ , where $I_{d}$ is the identity matrix of dimension d , accounts for the adjustment of the contact force magnitude, with ${\left\|f_{m}\right\|}^{2}I_{d}$ scaling the weight of $\Psi_{p}$ in all directions, and the outer product $f_{m}f_{m}^{⊤}$ influencing the update direction of the contact force. $\Psi_{c}$ represents the compensation term due to torque error, and its update strategy is as follows:

$$\begin{array}{c} \Psi_{c}\left(t\right)=\Psi_{c}\left(0\right)+\int_{0}^{t}\left(-\xi \Psi_{c}\left(u\right)+f_{m}\left(u\right)\times \tau_{m}\left(u\right)\right)du \end{array}$$ (5)

where $\Psi_{c}\left(0\right)$ represents the initial error matrix, and ξ controls the compensation rate. In Equation 5, the cross-product reflects the interaction between the contact force and contact moment.

Using an adaptive control method, the estimated value of the surface normal vector $s_{n}$ is gradually refined based on the velocity feedback from the end effector. This enables the decomposition of the contact force, allowing control over both the contact force in the normal direction and the tangential sliding force. The adaptive update strategy for the normal vector is formulated in Equation 6.

$$\begin{array}{c} s_{n}\left(t\right)=s_{n}\left(0\right)-\int_{0}^{t}\lambda G\left(s_{n}\left(u\right)\right)\Psi_{n}\left(u\right)s_{n}\left(u\right)du \end{array}$$ (6)

where $G\left(s_{n}\left(u\right)\right)=I_{d}-s_{n}\left(u\right)s_{n}^{⊤}\left(u\right)$ , and $s_{n}\left(0\right)$ represents the initial surface normal vector, which is set to [001] , the parameter λ adjusts the convergence rate of the estimation. The term $I_{d}-s_{n}s_{n}^{⊤}$ projects the vector onto the orthogonal plane of $s_{n}$ without altering the magnitude of the normal vector. The dynamic update of $\Psi_{n}$ is based on the motion of the end-effector, and its update strategy is as shown in Equation 7.

$$\begin{array}{c} \Psi_{n}\left(t\right)=\Psi_{n}\left(0\right)+\int_{0}^{t}\left(-\zeta \Psi_{n}\left(u\right)+Q\left({\dot{p}}_{e}\left(u\right)\right)\right)du \end{array}$$ (7)

where $\Psi_{n}\left(0\right)$ represents the initial state matrix for the normal vector estimate, initialized as a null matrix. The update rate is controlled by ζ , and the term $Q\left({\dot{p}}_{e}\left(u\right)\right)=\alpha \left({\dot{p}}_{e}\left(u\right)\right)\left({\dot{p}}_{e}\left(u\right)⊗{\dot{p}}_{e}\left(u\right)\right)$ generates a symmetric matrix through the outer product, reflecting both the magnitude and direction of the end-effector’s velocity. The normalization factor $\alpha \left({\dot{p}}_{e}\right)=\frac{1}{1+\|{\dot{p}}_{e}{\|}^{2}}$ ensures that updates remain stable and adaptive by preventing excessive adjustments at low velocities.

#### 3.3.2 Contact estimation-based transition velocity adjustment

When the robot reaches the target point without control, there is a possibility of direct contact between the end-effector and the contact point (Ma et al., 2021). Guaranteeing a stable critical contact force prevents excessive impact forces at the moment of physical interaction between the robot and the human. To address this issue, dynamic velocity adjustment is implemented to reduce the amplitude of oscillation, enhancing the overall stability and safety of the contact.

This adjustment not only facilitates real-time estimation of the robot’s normal direction and contact point but also helps prevent the up and down fluctuation caused by reverse compensation of the contact force. By incorporating dynamic velocity adjustment, the robot can ensure consistent and stable interactions with its environment, promoting improved accuracy and performance during the task (Michel et al., 2021).

In the process of task execution, dynamic velocity regulation is adopted to avoid the phenomenon of position and force instability at contact time. The velocity changes dynamically with the time of task execution, and is also limited by the distance between the end-effector and the target point. The relationship between time and velocity during task execution can be expressed as:

$$v=\left\{\begin{array}{c} \chi -\frac{1}{e^{\theta \left(1-t\right)}+1},t<\varepsilon \\ \chi e^{-k\theta {\left(t-\chi \right)}^{2}},t\geq \varepsilon \end{array}\right.$$ (8)

The overall strategy is divided into two phases: the first phase achieves progressive deceleration to avoid direct collision, while the second phase performs velocity fine-tuning to ensure stable contact. Here, χ denotes the velocity upper bound, θ controls the curvature of velocity reduction, and k represents the weighting factor for velocity smoothing. This factor balances the rate of velocity change between the two intersecting segment functions, with ε defining the switching timepoint. At t=ε , the intersection of both functions guarantees velocity continuity, enabling smooth transitions and preventing system instability induced by abrupt acceleration/deceleration.

### 3.4 Contact force error-aware hybrid control

#### 3.4.1 Impedance control based on contact point position

At the control level, the dynamic model of a rigid robot with n degrees of freedom can be expressed in Equation 9.

$$\begin{array}{c} M\left(q\right)\ddot{q}+C\left(q,\dot{q}\right)+g\left(q\right)=\tau_{c}+\tau_{\mathrm{ext}} \end{array}$$ (9)

where $q\in R^{n}$ , $\dot{q}$ , and $\ddot{q}$ denote the joint position, velocity, and acceleration vectors, respectively; M(q) is the joint-space inertia matrix; $C\left(q,\dot{q}\right)$ represents the Coriolis and centrifugal terms; g(q) denotes the gravity vector; $\tau_{c}$ is the commanded control torque; and $\tau_{\mathrm{ext}}$ is the external torque caused by physical interaction with the patient or silicone model.

The dynamic modelling of the end-effector is represented in the form of a mass-spring-damper system:

$$\begin{array}{c} \Gamma \ddot{x}=K△x-D\dot{x}+F_{\mathrm{ext}} \end{array}$$ (10)

where Γ is the virtual spring-mass matrix of the physical system, K denotes the stiffness matrix, D corresponds to the critical damping matrix, and $F_{\mathrm{ext}}$ is the external forces. Through the above system, the mechanical characteristics of the robot can be adjusted to meet the requirements of different interaction modes and controls.

In our framework, the time-varying stiffness parameter values in Equation 10 are associated with the are associated with the extent of dispersion among the trajectory points after generalization. When the trajectory points are densely packed within a given time interval, signifying a longer execution time for that trajectory, a higher stiffness value is necessary to ensure stable and precise compliance control. The formula for the time-varying stiffness is as follows:

$$\begin{array}{c} K=K_{\min}+\frac{1}{1+e^{-r\left(\delta_{t}-\delta_{0}\right)}}\left(K_{\max}-K_{\min}\right) \end{array}$$ (11)

In the Equation 11, the $K_{\max}$ and $K_{\min}$ denote the maximum and minimum stiffness values, respectively, that allow for the smooth execution of the task without triggering a collision. This equation ensures that the stiffness values remain within the specified range and can adapt over time according to the requirements of the task. By limiting the stiffness within the maximum and minimum values, the robot’s compliance control can be effectively regulated to ensure safe and accurate interactions during the execution of the trajectory.

The impedance parameters are scheduled according to the task phase and the local characteristics of the generalized DMP trajectory. Specifically, the density of trajectory points is used as an indicator of local execution demand. A denser point distribution usually corresponds to a slower or more precise therapeutic motion segment, where a relatively higher stiffness is required to maintain motion accuracy and stable force regulation. In contrast, during approach, departure, or uncertain contact transition phases, a lower stiffness is assigned to enhance compliance and reduce the risk of uncomfortable interaction. The stiffness is constrained within $\left[K_{\min},K_{\max}\right]$ , and the damping is set according to the critical damping condition to avoid underdamped oscillation.

#### 3.4.2 Force adjustment strategy based on surface normal

When the robot’s end-effector comes into contact with the skin, the presence of significant pulse forces can lead to discomfort for the patient, reduce the effectiveness of treatment, and in severe cases, even cause harm to the human body. To address this, a force regulation strategy is implemented, primarily based on establishing a mathematical model that relates the feedback force to the position.

Considering the engagement with human soft tissues, the Kelvin-Voigt viscoelastic model has been integrated. This model allows for a better adaptation to the surface deformation of the human body by taking into account the feedback from the interactions between the robot and the soft tissues. The external force can be formulated in Equation 12

$$\begin{array}{c} F_{\mathrm{est}}=\Phi \left(F_{f}\right)+K_{m}\left({\hat{p}}_{r}-x\right)+D_{m}\left(\dot{x}\right) \end{array}$$ (12)

where $\Phi \left(F_{f}\right)$ is the composite function of the feedback force $F_{f}$ , which is estimated to be:

$$\begin{array}{c} \Phi \left(F_{f}\right)=\epsilon \hat{F_{f}}+\eta \int_{t}^{t-T}\hat{F_{f}}dt \end{array}$$ (13)

In Equation 13, the ϵ and η are scale factors of control force compensation efficiency, $K_{m}$ and $D_{m}$ are parameter values of the KV model, and T represents the selected time window. In the process of robot intervention, the calculation error and scale factor are the key factors to determine the appropriate compensation strategy to ensure the precision of force control.

#### 3.4.3 Stability and safety considerations of variable impedance control

Variable impedance control may introduce instability if stiffness or damping changes abruptly during physical interaction. To mitigate this risk, the proposed controller uses bounded impedance parameters, continuous velocity regulation, and force-feedback compensation. First, the stiffness is constrained within $\left[K_{\min},K_{\max}\right]$ , which prevents the controller from becoming excessively rigid during contact. Second, the damping term is adjusted with respect to the stiffness to reduce oscillation. Third, the dynamic velocity strategy provides a continuous transition during the approach-contact phase, avoiding sudden acceleration or deceleration. Finally, the contact force error is compensated in real time along the estimated surface normal, which reduces persistent force deviation and suppresses impulsive interaction. These mechanisms improve the practical stability of the system during contact-rich physiotherapy tasks.

## 4 Experimental verification

The experiments aim to evaluate the control-level performance of the proposed framework rather than clinical therapeutic efficacy. Therefore, the evaluation focuses on contact safety, force regulation accuracy, motion stability, and task adaptability. The proposed method is compared with fixed-speed contact strategies, no-force-compensation control, and fixed-stiffness impedance control.

**FIGURE 5:**
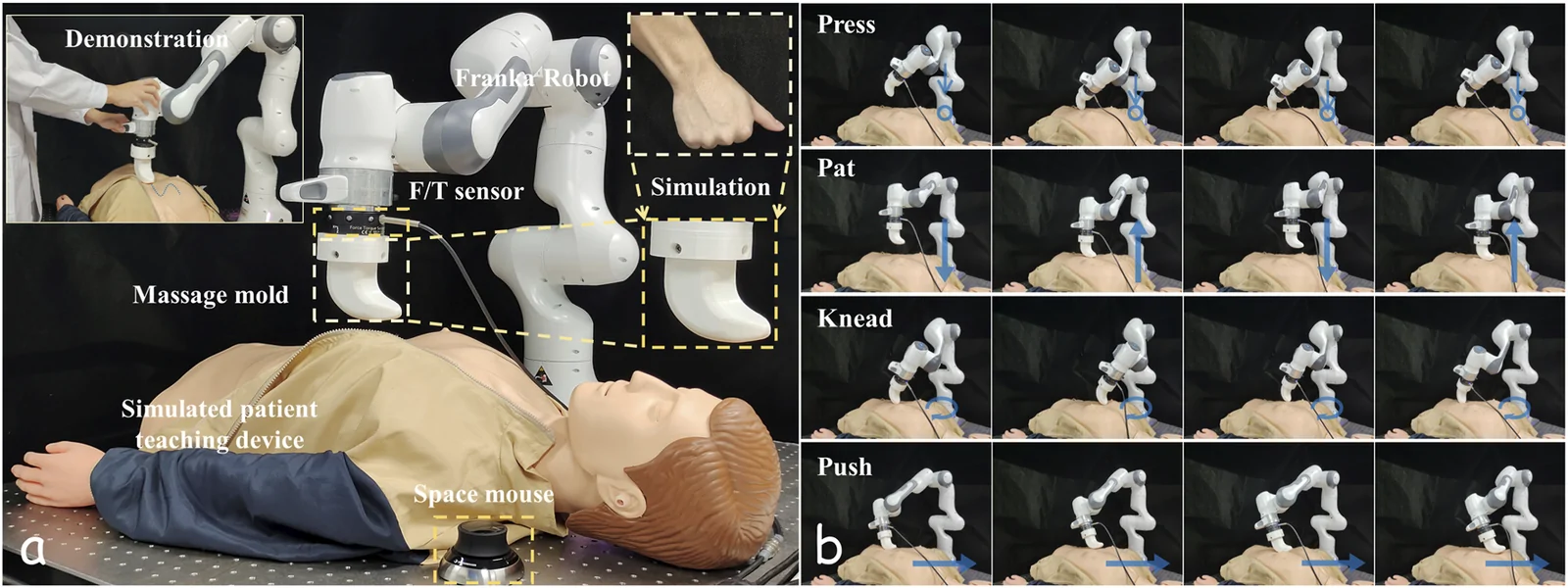
Experimental setup and four massage skills validated on the silicone abdominal model. **(a)** Robotic physiotherapy platform with a Franka Emika Panda robot, a six-axis force/torque sensor, and a thumb-inspired end-effector. **(b)** Four massage skills, including pressing, patting, kneading, and pushing.

To validate the proposed approach in this study, the robot was demonstrated using abdominal massage skills and back physiotherapy skills. The demonstration learning process, depicted in Figure 5, involved manually guiding the robot to acquire physical therapy skills. This setup allowed for effective learning and control of the robot’s interactions, ensuring accurate and safe performance of massage techniques. In the experiments, an abdominal CPR training manikin served as the experimental model. This standardized silicone model, designed for emergency medical training, features a soft external silicone structure that primarily simulates human thoracic compression characteristics. Its surface exhibits measurable flexibility and compressibility, approximating certain physical properties of human soft tissues.

During the data acquisition process, the therapist guides and demonstrates massage techniques by manipulating the robot’s end-effector. Simultaneously, the three-dimensional trajectory of the robot’s end-effector in Cartesian space is recorded. The training dataset consists of three massage methods: patting, rubbing, and pushing. To improve adaptation, we initially generate three demonstrated trajectories, and then we use the spatial mouse to determine the start and end points of the generalized trajectory in the task space. The massage skill encoded in the training dataset can be adapted to new behavior. As shown in Figure 5, the robot performs four abdominal massage skills on a silicone model.

During training, the therapist guided the robot end-effector through kinesthetic teaching to demonstrate different massage skills. The Cartesian position and velocity of the end-effector were recorded at a fixed sampling frequency. Each demonstrated trajectory was preprocessed by temporal normalization and smoothing. Then, the DMP parameters were learned by fitting the nonlinear forcing term with radial basis functions. Each skill was stored as an individual primitive in the physiotherapy skill library. During execution, the operator selected a skill and specified the start and goal points on the target contact region. The corresponding DMP generated the nominal reference trajectory, which was further adjusted online according to the estimated contact point, surface normal, and force feedback.

### 4.1 Dynamic contact strategy

**FIGURE 6:**
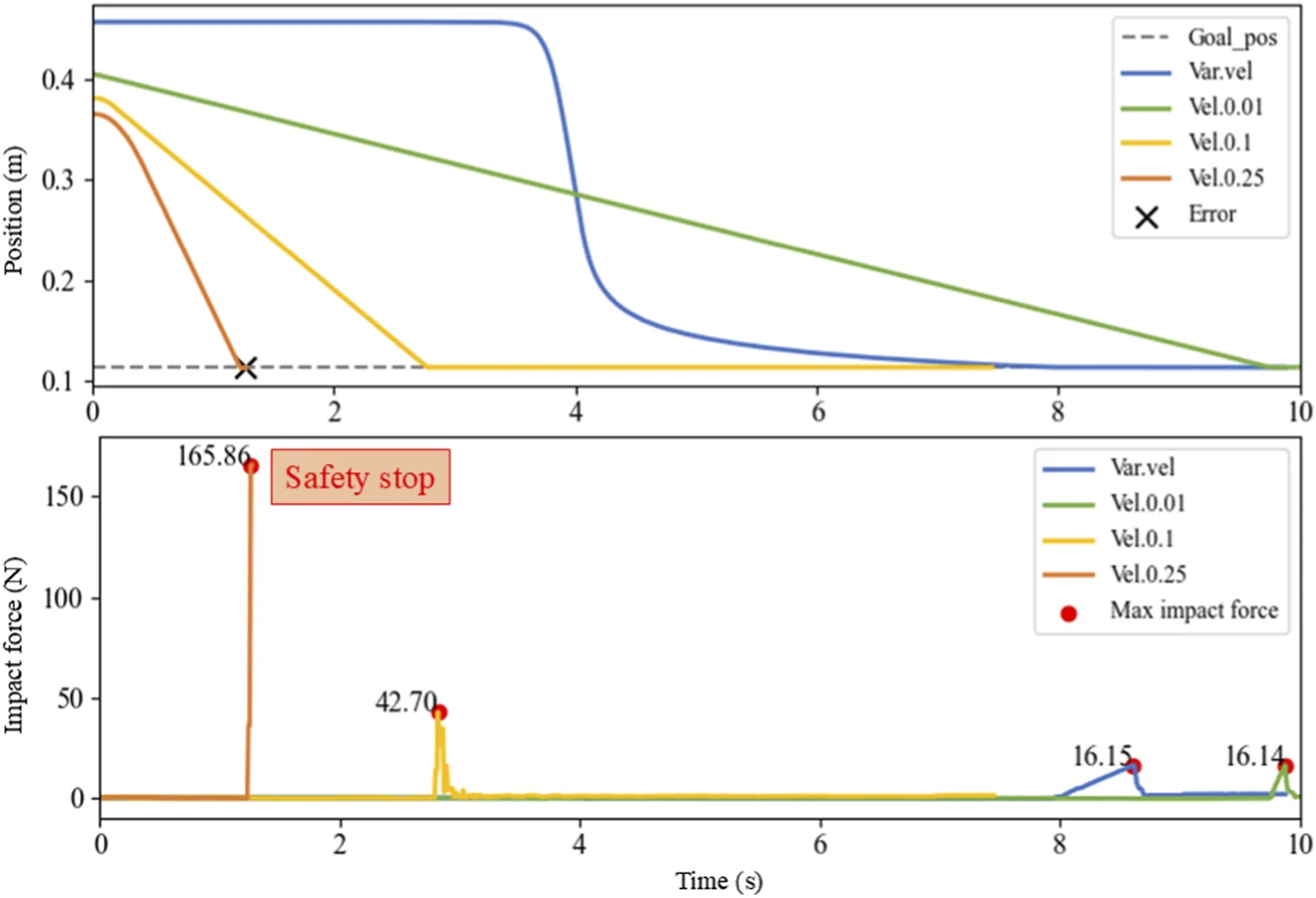
Comparison of contact transition performance under fixed-velocity and dynamic-velocity strategies.

A dynamic adjustment strategy was developed and tested on four physiotherapy skills. During the execution of robot tasks, achieving stable and efficient contact with the target surface is crucial. As shown in Figure 6 and Table 1, the high-speed strategy caused a peak impact force of 165.86 N , and the fixed velocity of 0.1 m/s still generated a peak force higher than 40N . In contrast, the proposed dynamic velocity strategy achieved stable contact transition without excessive impact. The value of ξ in Equation 8 was set at 0.25m/s , corresponding to a high-velocity setting suitable for the robot’s safe operation. However, when performing the contact task at maximum velocity, the robot reaches the contact surface with excessive velocity, resulting in an impact force of up to 165.86N . In real massage experiments, such instantaneous impact could potentially harm the patient, highlighting the need for velocity adjustment based on contact. Testing at 0.1m/s still resulted in an instantaneous contact force exceeding 40N . On the other hand, selecting a minimum velocity of 0.001m/s ensures stable contact force but leads to a significant half-minute time loss. In practical scenarios, this is unnecessary and reduces patient satisfaction. Our proposed variable strategy not only ensures stable contact but also eliminates the interference of real-time starting positions, making it safer and more practical.

**TABLE 1:** Quantitative comparison of control performance.

Evaluation item	Baseline	Proposed method	Metric
Contact approach	Fixed velocity 0.25 m/s	Dynamic velocity	Reduce peak impact force
Contact approach	Fixed velocity 0.1 m/s	Dynamic velocity	Reduce peak impact force
Force regulation	No compensation	Force compensation	RMSE reduced 52.69%
Hybrid control	Fixed low/high stiffness	Variable stiffness	Improved tracking stability without triggering collision stop
Human-subject back test	Feasibility only	Proposed framework	Stable execution of knead, pat, push, and press skills

### 4.2 Contact point and normal estimation

When dealing with non-conventionally flat contact surfaces, such as the human body, directly sending message commands to the robot can lead to unexpected collisions, causing the robot to stop. To address this issue, the framework incorporates contact points and normal estimation.

The contact point can be accurately estimated based on the interactive contact force by the torque sensor. This adaptive adjustment enables the robot to effectively accommodate various gestures during the physiotherapy process. Unlike traditional methods that rely on fixed contact points, this approach allows for more flexibility and adaptability.

**FIGURE 7:**
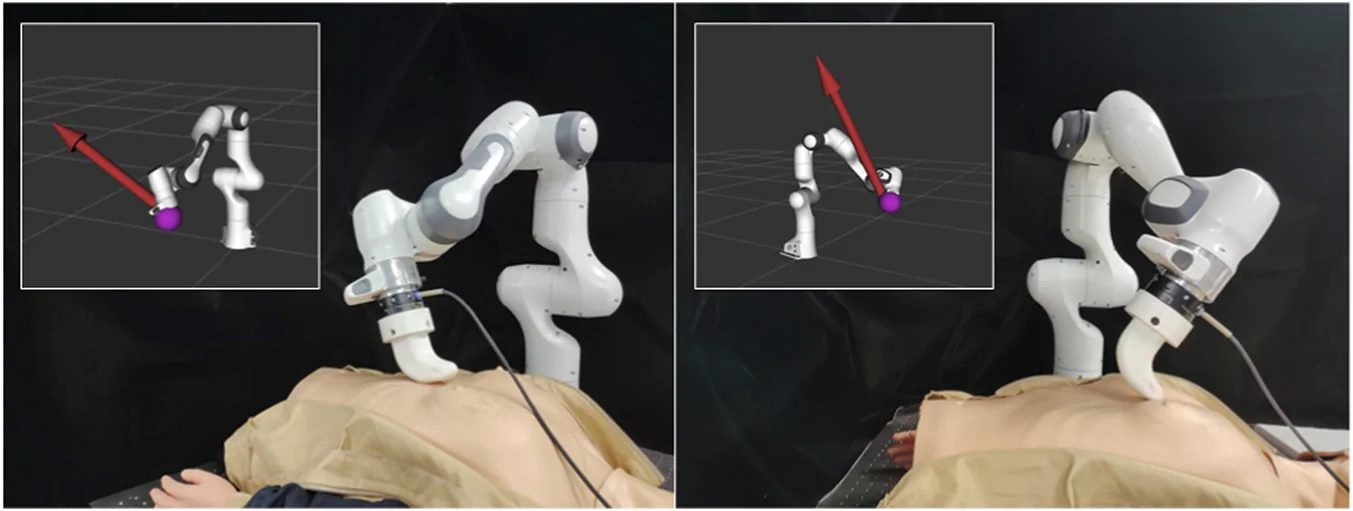
Real-time contact point and surface normal estimation under two end-effector poses. The purple sphere denotes the estimated contact point, and the red arrow denotes the estimated surface normal.

The normal estimation component is capable of adapting to surfaces with small curvature. It determines an appropriate normal adjustment strategy based on the external force applied to the contact point. By combining this strategy with the robot’s rotation, the robot dynamically adjusts itself to align with the normal plane of the contact surface where the contact point is situated. This ensures that the robot maintains a perpendicular relationship with the contact surface, regardless of its curvature, as shown in Figure 7.

### 4.3 Force error compensation strategy

To evaluate the stability of the force feedback controller, we conducted the following experiments. The robot was programmed to apply a force of 10+2⁡sin(t)N and perform a pressing test on a silicone plate. By observing the changes in force tracking during the robot’s execution, we aimed to achieve more stable force variations and reduce tracking errors. When the robot interacts with a soft tissue, the dynamic tracking compensation strategy exhibits a favorable response. It allows for quick stabilization after contact and achieves the desired force. On the other hand, if no compensating controller is implemented, a persistent and stable error can persist for a certain period of time.

**FIGURE 8:**
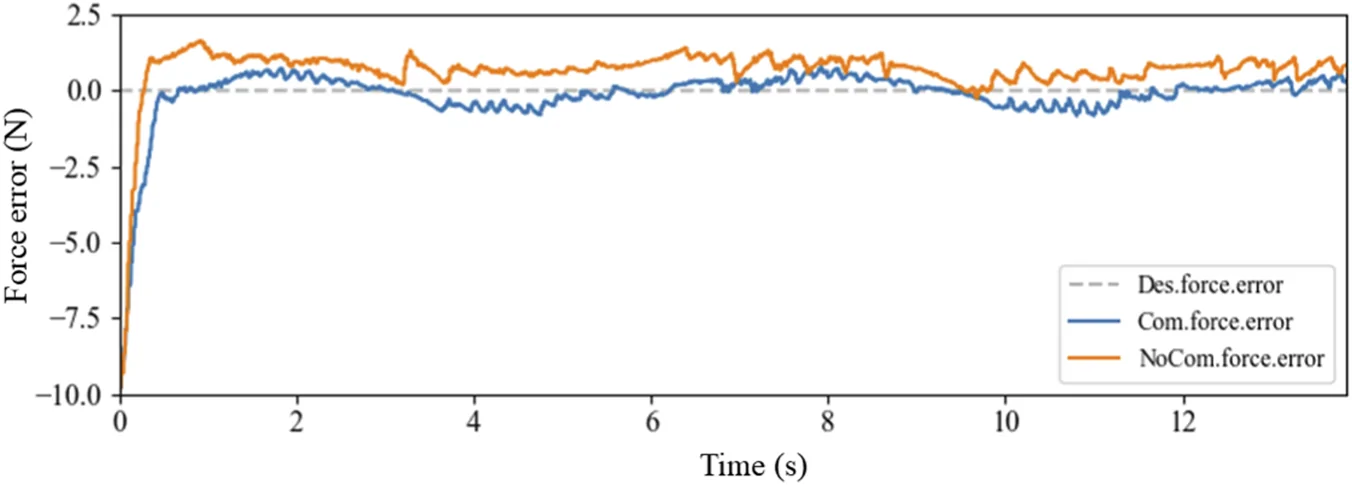
Force tracking error with and without force compensation.

As shown in Figure 8, force compensation substantially reduces the steady-state force error after contact. Without compensation, the force error remains biased for a longer duration, whereas the proposed compensation strategy allows the force to converge faster to the desired profile.

We computed the root mean square error (RMSE) (Hodson, 2022) between the force information obtained using the compensating controller strategy and the desired force. The calculation results are as follows: the RMSE of the compensating controller is 0.395N , which is 52.69% more accurate than the other strategy, with an RMSE of 0.835N . These results demonstrate the advantages and necessity of implementing the compensating controller in various aspects, as evidenced by the error calculations.

### 4.4 Hybrid control strategy

According to the DMPs, we conducted experiments involving beating tasks on a deformable object under conditions of low, high, and variable stiffness. When selecting a stiffness strategy, it is important to fulfil both the requirements of motion planning and the application of the expected force during contact. After extensive testing, we established a minimum stiffness value of 100(Ns/m) and a maximum stiffness value of 1000(Ns/m) . By considering the density of track points obtained from real-time DMP training, we derived a set of stiffness configurations during runtime.

The stiffness range of 100−1000Ns/m was selected based on preliminary safety tests on the silicone model. The lower bound of 100 Ns/m provided sufficient compliance during approach and contact transition, while smaller values led to poor trajectory tracking and insufficient force regulation. The upper bound of 1000Ns/m improved motion accuracy during precise therapeutic motion, while larger values increased the risk of rigid contact and collision-triggered robot stop. Therefore, this range was used as a practical trade-off between compliance, tracking accuracy, and contact safety.

**FIGURE 9:**
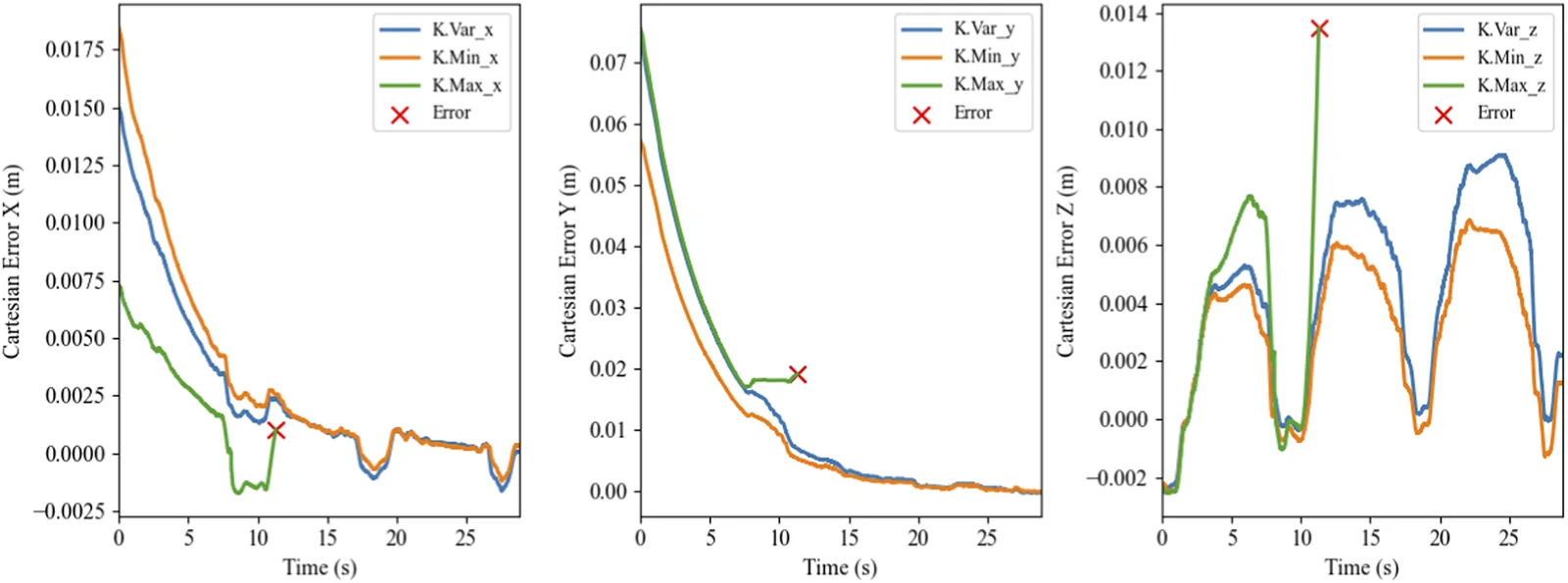
Position responses under fixed high-stiffness, fixed low-stiffness, and variable-stiffness strategies.

The variable stiffness method demonstrates the best adaptability when both motion and force are applied to the Z-axis, as shown in Figure 9. From the figure, it can be observed that different strategies can successfully complete the task under both low and variable stiffness conditions. Furthermore, the variable stiffness strategy exhibits superior stability compared to the fixed stiffness strategy.

### 4.5 The patient’s back physiotherapy

The back physiotherapy experiment was conducted on one healthy volunteer. The recruited volunteer was male and 24 years old. The inclusion criteria were: no known musculoskeletal disorders, no back skin injury, no acute pain, and no history of spinal surgery. The participant provided written informed consent before the experiment. The experiment involved only low-risk contact operations on the dorsal surface and did not include invasive procedures or medical intervention. It should be noted that the current human-subject experiment was intended as a preliminary feasibility and safety validation of the proposed robotic physiotherapy framework, cannot be interpreted as clinical evidence.

**FIGURE 10:**
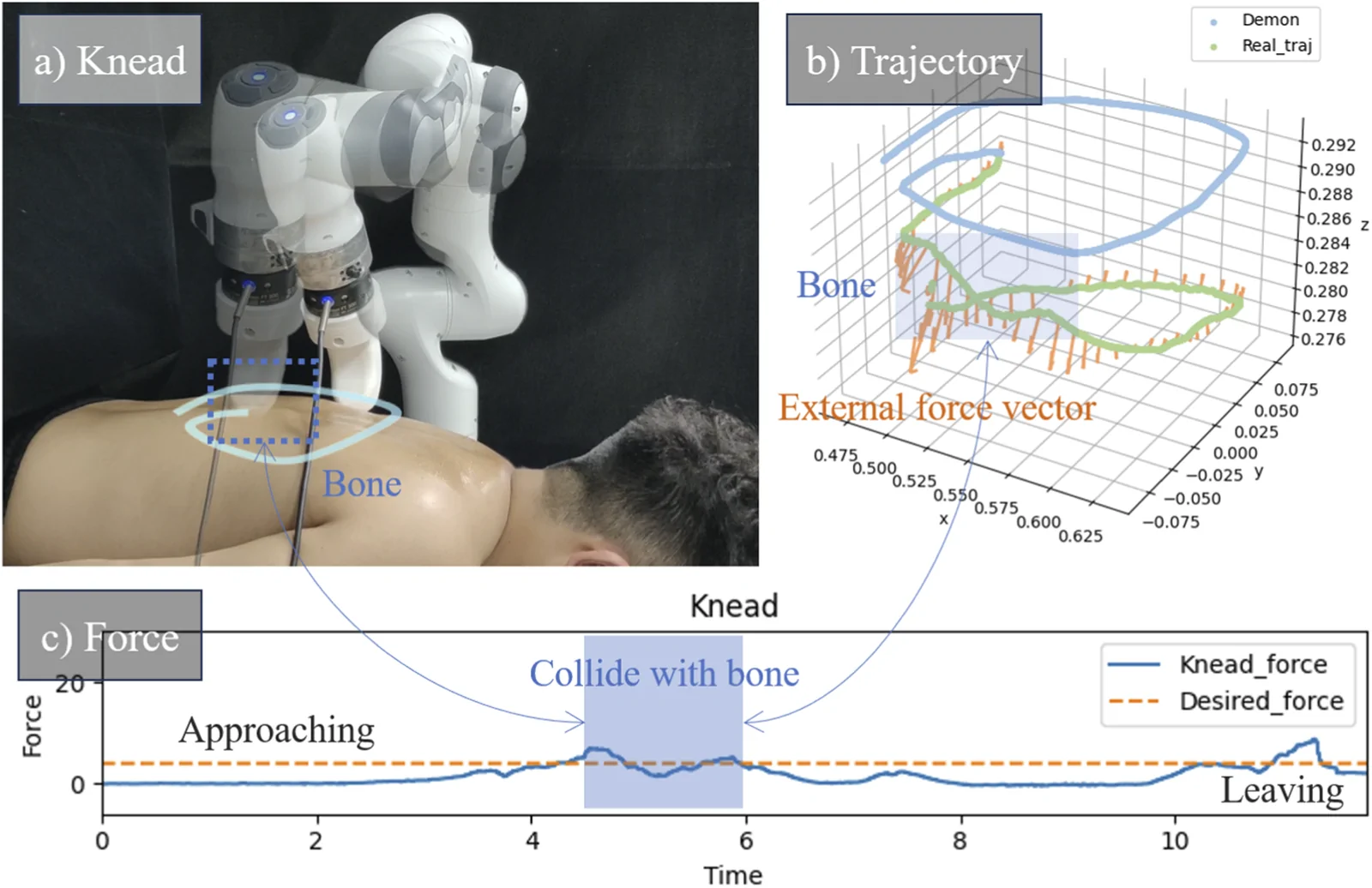
Back kneading experiment on a healthy volunteer. **(a)** Experimental scene. **(b)** Demonstrated and executed trajectories. **(c)** Force profile during kneading.

We have verified the established massage skill models in a real-life experimental scenario, as depicted in Figure 10. Utilizing the tip of the end-effector to perform a kneading operation akin to circular motion, the task necessitates the application of the desired force during concurrent changes in position and posture. Unlike planar motion, when the robot executes on the human back, particularly at the spine, skeletal collisions can lead to fluctuations in the external force and the actual position. The proposed contact-aware control strategy adapts to these variations by adjusting the local surface normal, contact force, and impedance parameters online. Our method is adept at compliant control, preventing abrupt counteractions, thereby improving contact comfort and execution stability.

**FIGURE 11:**
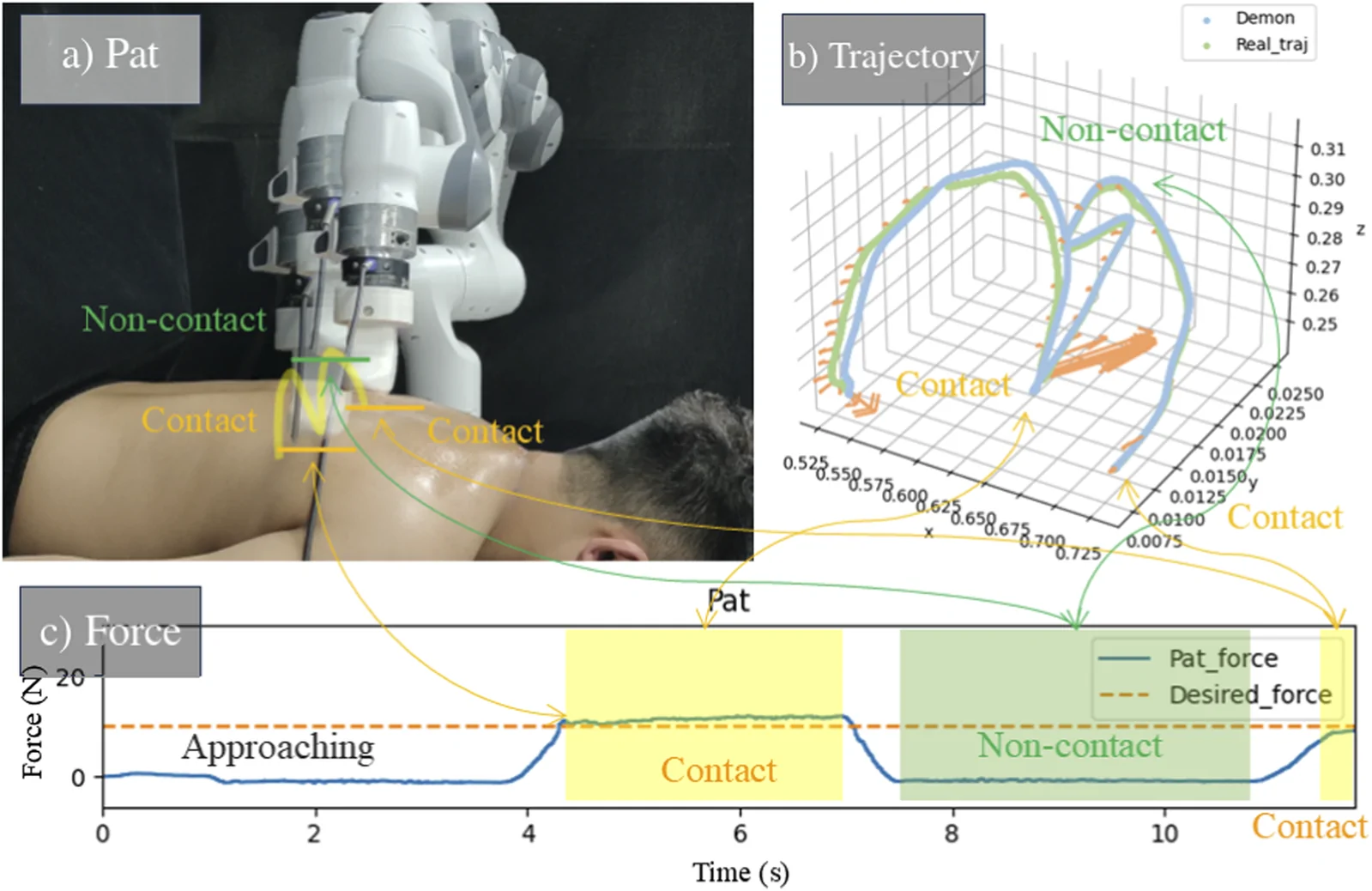
Back patting experiment. **(a)** Patting execution. **(b)** Trajectory comparison. **(c)** Contact and non-contact phases during patting.

During the execution of the pat task, the bend of the end-effector imitates the human palm, as depicted in Figure 11. It is observable from the figure that our method maintains a stable force upon contact, demonstrates high efficiency in transitioning between contact and non-contact states, and also achieves a high degree of positional and force accuracy.

**FIGURE 12:**
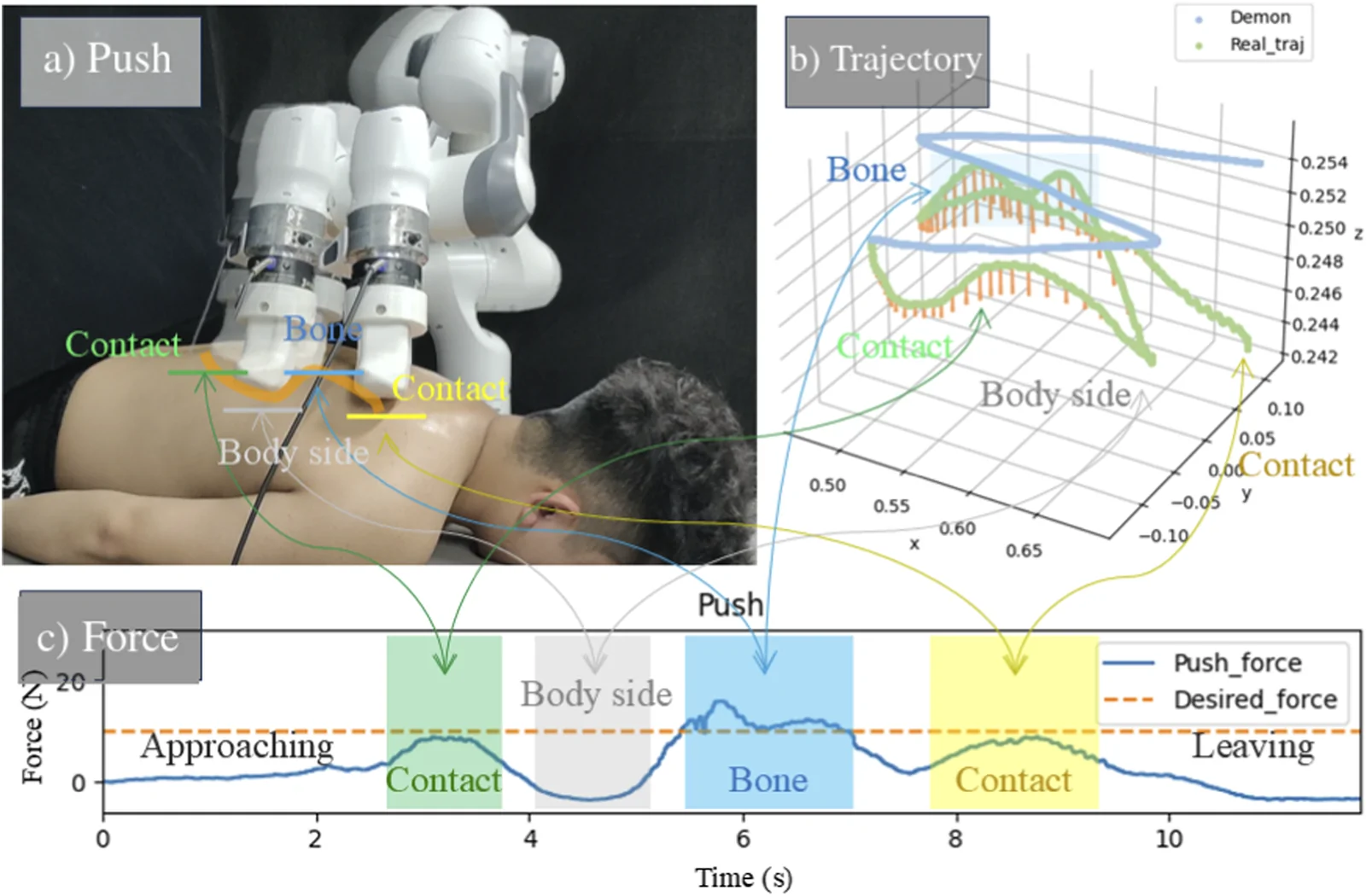
Back pushing experiment. **(a)** Pushing execution. **(b)** Trajectory and contact region. **(c)** Force response when passing different back regions.

In the process of pushing skill as illustrated in Figure 12, after contact is made, the massage may be performed on the side of the body. In our method, when a significant force error is encountered during the execution, it can affect the task gain and initiate task-driven adjustments. Subsequently, a compliant control is employed to navigate past the spine, thereby completing both the force and positional tasks.

**FIGURE 13:**
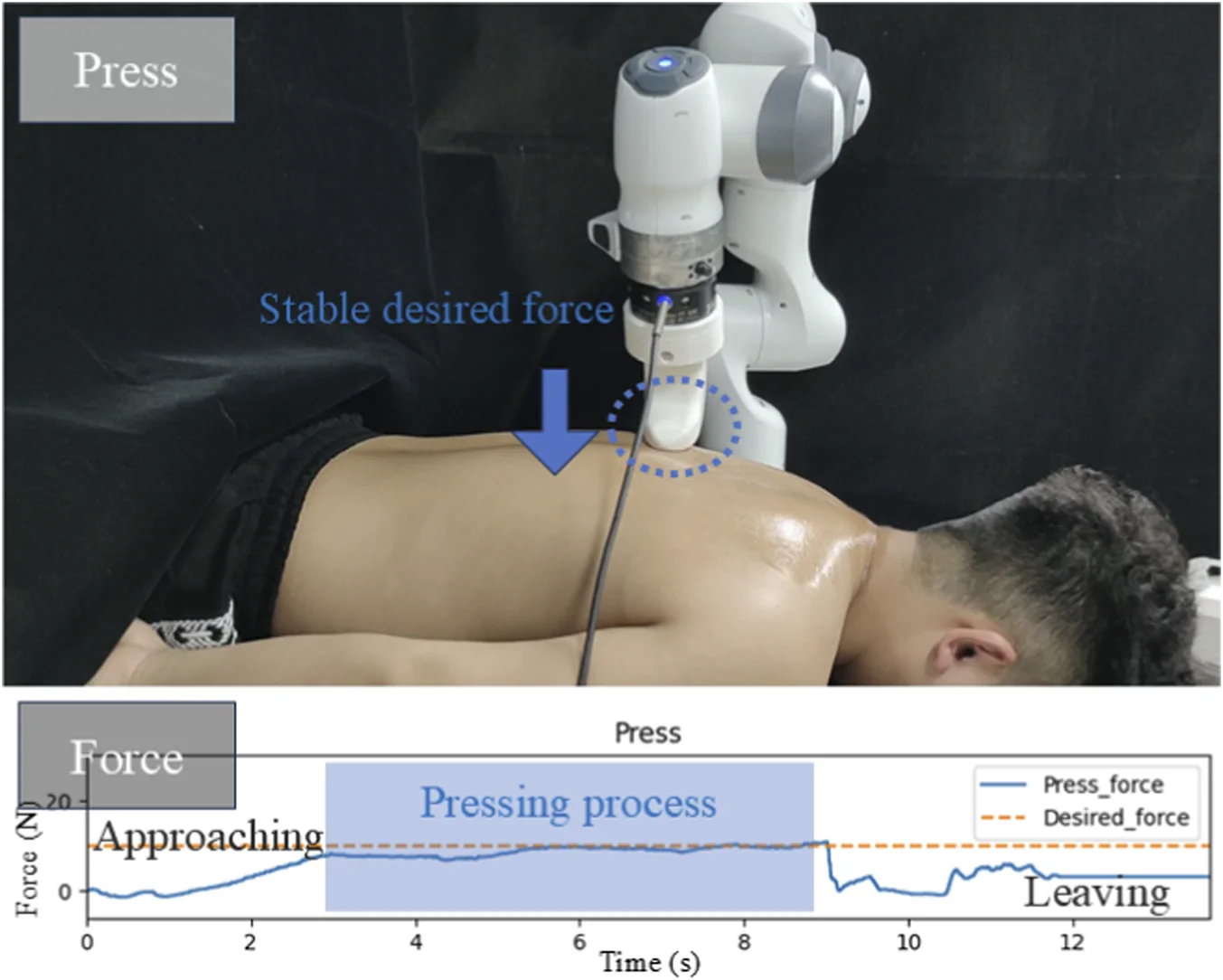
Back acupoint pressing experiment and corresponding force response.

The task of acupoint pressing, as depicted in Figure 13, is divided into three phases: the approach phase, the pressing phase, and the departure phase. During the pressing process, there is a high degree of precision in force application. Moreover, due to the variations in the body surface height caused by the subject’s breathing during the actual pressing process, our method is still capable of overcoming this uncertainty and achieving satisfactory experimental results.

This study involved only basic contact operations on the dorsal surface of one healthy volunteer, constituting a low-risk procedure without invasive techniques or medical interventions. The participant provided written informed consent. According to our institution’s guidelines, such low-risk experiments do not require formal IRB approval and were instead approved internally within the laboratory. (Approval ID: LabEthics-2025-001).

## 5 Conclusion and future work

This paper introduces a contact-aware robotic physiotherapy framework that integrates task-space skill generalization, contact point and surface normal estimation, dynamic velocity adjustment, force-error compensation, and bounded variable impedance control for stable constant-force robotic therapy through human demonstrations. In the proposed framework, therapist-guided demonstrations are encoded as DMPs in the robot Cartesian task space, enabling typical physiotherapy skills to be generalized to new start and goal points rather than performing cross-embodiment skill transfer. The framework uses the trained model and parameters to generate nominal reference trajectories, while force/torque feedback is used to update the local contact frame and adjust the robot’s motion and force output online. The stiffness parameters are scheduled according to the skill phase, the density of the generalized DMP trajectory, and the contact state, thereby balancing motion accuracy and contact compliance.

The framework considers motion effectiveness, steady force application, and smooth control for robotic skills. Compared with fixed-speed contact, no-force-compensation control, and fixed-stiffness impedance strategies, the proposed method improves contact transition safety, force regulation accuracy, and motion-force coordination. In the experiments, the dynamic contact strategy avoided the excessive 165.86 N peak impact observed under high-speed contact, and the force compensation strategy reduced the force-tracking RMSE from 0.835 N to 0.395 N , corresponding to a 52.69% improvement. In addition, our method has been tested with abdominal massage on a silicone model and a preliminary healthy-volunteer back physiotherapy experiment. The results show that it has good adaptability and stability at the control-performance level. This research provides valuable insights into robots’ application and integration in the healthcare field, expanding their potential use beyond the industrial domain.

It should be noted that the current human-subject experiment was designed as a preliminary feasibility and safety validation rather than a clinical efficacy study with statistical generalizability. Future work includes conducting real-time DMP training, incorporating probabilistic movement primitives or other statistical learning methods to fuse multiple therapist demonstrations, addressing conflicts between variable impedance control and force compensation, and enhancing the framework’s generalization capability. Further studies will also recruit more volunteers and introduce subjective comfort assessment and clinical evaluation protocols to verify the therapeutic effectiveness of the proposed robotic physiotherapy system.

Although the proposed bounded impedance and smooth velocity transition improved practical contact stability, this work does not provide a formal passivity or Lyapunov stability proof for the complete variable impedance system. Future work will incorporate passivity observers/controllers or energy-tank-based mechanisms to provide formal stability guarantees during continuous human-robot contact.

## Data Availability

The raw data supporting the conclusions of this article will be made available by the authors, without undue reservation.
